# Nursing care time and quality indicators for the ICU: correlation analysis

**DOI:** 10.1186/cc12629

**Published:** 2013-06-19

**Authors:** PC Garcia, FMT Fugulin

**Affiliations:** 1 Hospital Universitário da Universidade de São Paulo, Cidade Universitária, São Paulo, SP, Brazil; 2 Escola de Enfermagem, Universidade de São Paulo, SP, Brazil

## Introduction

The objective of this exploratory, retrospective, quantitative study was to analyze the time spent by the nursing staff to assist patients in an adult ICU (AICU) of the University Hospital, University of São Paulo (UH-USP) and verify its correlation with quality care indicators.

## Methods

This research started on 1 January 2008 until 31 December 2009. Data were collected from the administration tools used by the head nursing staff of the unit. Analysis of the average length of time in relation to the average length of time required by patients was performed using the paired *t *test. The correlation coefficient was used to verify the correlation of the average length of care time given to patients in the AICU with the quality indicator incidence.

## Results

The average length of time regarding assistance for patients, in the analyzed period, accounted for 14 hours, of which 31% were performed by nurses and 69% by technicians/nursing assistants. The hospitalized patients required approximately 16 hours of care. The application of statistical tests showed that the differences found between the hours of assistance given by the nursing staff and those required by patients was significant (*P <*0.001), suggestive of the heavy workload for the nurses in the AICU. The correlational analysis between the length of time of nursing care given by nurses and the quality indicator incidence of accidental extubation evidences Pearson's correlation coefficient (*r *= -0.454), with *P *= 0.026, indicating negative linearity between variables, which allowed us to infer that the incidence of accidental extubation decreases with increasing nursing care time given by nurses.

## Conclusion

The results revealed that the average hours of nursing care for patients of the AICU were lower than those recommended by the official Brazilian Agencies. The average time of care required by patients hospitalized in this unit was higher than that recommended by ANVISA and lower than that established by COFEN Resolution Nr. 293/04. This indicates that the quantitative assessment of nursing staff in ICUs requires prior knowledge of the users' healthcare demands and not only the use of parameters indicated by official agencies, since this procedure may cause an overdimensioning or underdimensioning of nursing professionals. The results of this study showed the influence of nursing care time provided by nurses in the outcome of care given to patients assisted in the AICU. The addition of more evidence may help to demonstrate the impact of nursing care time in healthcare results and patient safety.

